# Resisting hostility generated by terror: An agent-based study

**DOI:** 10.1371/journal.pone.0209907

**Published:** 2019-01-14

**Authors:** Sylvie Huet, Guillaume Deffuant, Armelle Nugier, Michel Streith, Serge Guimond

**Affiliations:** 1 Laboratory of Engineering for Complex Systems (LISC), Irstea, Aubière, France; 2 Laboratory of Social and Cognitive Psychology (LAPSCO), UCA, Clermont-Ferrand, France; Centre National de la Recherche Scientifique, FRANCE

## Abstract

We propose an agent-based model leading to a decrease or an increase of hostility between agents after a major cultural threat such as a terrorist attack. The model is inspired from the Terror Management Theory and the Social Judgement Theory. An agent has a cultural identity defined through its acceptance segments about each of three different cultural worldviews (i.e., Atheist, Muslim, Christian) of the considered society. An agent’s acceptance segment is composed from its acceptable positions toward a cultural worldview, including its most acceptable position. An agent forms an attitude about another agent depending on the similarity between their cultural identities. When a terrorist attack is perpetrated in the name of an extreme cultural identity, the negatively perceived agents from this extreme cultural identity point of view tend to decrease the width of their acceptance segments in order to differentiate themselves more from the threatening cultural identity. We generated a set of populations with cultural identities compatible with data from a survey on attitudes among a large sample representative of the population of France; we then simulated the reaction of these agents facing a terrorist attack from Muslim extremists. For most populations, the average attitude toward Muslims becomes more negative. However, for some specific populations, we noticed the opposite effect as the average attitude of the population toward Muslims becomes less negative. In these populations, the Muslim agents strongly differentiate themselves from the terrorists’ extreme cultural identity, and the other agents are aware of these changes. These reactions are due to particular properties of their cultural identities that are identified in this paper.

## Introduction

Terrorist attacks perpetrated by religious extremists have been experienced at an alarming rate in recent years. Although political violence can be related to a wide variety of issues, from the extreme-left to the extreme-right, data from the National Consortium for the Study of Terrorism and Response to Terrorism (START) from the University of Maryland (http://www.start.umd.edu/) indicates: “In comparison to the 2000s, there was a sharp decline in the proportion of terrorist attacks carried out by left-wing, environmentalist extremists during the first seven years of the 2010s (from 64% to 12%). At the same time, there was a sharp increase in the proportion of attacks carried out by right-wing extremists (from 6% to 35%) and religious extremists (from 9% to 53%) in the United States”.

The top perpetrator groups of these attacks were Jihadi-inspired groups and their attacks were also by far the most lethal in terms of the number of deaths. Worldwide, and for the year 2017 alone, the data indicate that ISIL (Islamic State of Iraq and the Levant) was the top perpetrator group with 1321 attacks followed by the Taliban with 907 attacks. Because major cities in the West were hit during the 2000s, Governments of France, the United States, the United Kingdom and many others quickly developed security measures and policies designed to stop violence perpetrated by religious extremists. A strong concern, motivating this research, is also the growing hostility between communities that may result from these attacks.

Our approach is inspired by the Terror Management Theory (TMT) [1]. In TMT, cultural worldviews, defined as “shared conceptions of reality” [2, 3], are an important defense mechanism allowing people to cope with existential threats. This is why people are motivated to maintain faith in them. TMT has shown that a death fatality reminder of any kind, such as for instance inviting the subject to imagine their own burial ceremony, is a cultural threat or a self-worth threat. Such a threat generally leads to increasing hostility toward minorities in order to defend one’s cultural worldviews [4, 5]. In case of death fatality caused by terrorist attacks, the growing hostility particularly targets those who can be judged to bear resemblance with the terrorism perpetrators (i.e. identified as supporting the same cultural worldview than the one supported by terrorists). However, recent research has shown that “increased prejudice and hostility are not an inevitable response to existential threat” [6]. Some cultural properties, when simultaneously becoming salient with the threat, increase the perceived similarity of members of different groups and protect them against an increase in intergroup hostility [6, 7]. Understanding when and why people react to a cultural or collective threat one way or the other is a basic problem having widespread theoretical and practical implications. We propose a new agent based model aiming at addressing this question.

An agent-based model of culture dynamics was seminally introduced by Axelrod [8]. The Axelrod model represents a culture as a set of traits and changes a cultural trait not shared by two agents into a shared one, with a probability depending on their level of shared properties. Several variants have been studied [9], also introducing a process that leads traits to become more different [8, 10–13] instead of being shared. However, none of these models starts from agents’ acceptable positions about a culture to define the attitudes of the agents about each other or models the impact of the rejection of an existential threat on their acceptable positions. This is also generally the case of opinion dynamics models, although some of them include the possibility of rejecting another’s opinion instead conforming [12] or incorporate some rules for the evolution of self-worth [14, 15].

In our model, an agent has a cultural identity defined by an acceptance segment corresponding to its acceptable positions for each of the main cultural worldviews available in its environment. The model defines also the attitude of an agent about another depending on the similarity of their cultural identities. We assume that a terrorist attack is perpetrated by agents with extreme cultural identities. These agents are perceived to be a threat by some agents, leading them to restrain their acceptance segments. These changes in acceptance segments modify the attitudes that the agents have about each other.

We generate a set of populations of agents with cultural identities leading to attitudes about each other that are compatible with data from a representative survey on groups’ attitudes conducted in France in 2014 [16]; we then submit these populations to a virtual terrorist attack from Muslim extremists. We generally observe an increase in hostility against Muslims except in particular cases where, to the contrary, the hostility decreases. We study the evolution of the population in relation to the agents’ initial cultural identities and propose some explanations for these variations.

The next section presents the model as well as the material. Section 2 shows how the model is initialized and parameterized. Section 3 gives details about the evolutions and the related cultural properties. Finally, we conclude this paper and discuss our results.

## Materials and method

The first subsection presents at first the model, namely how we represent cultural identities through and how they change after a terrorist attack. The second subsection describes how 120 initial populations of agents have been built to study by simulation the evolution of attitudes toward Muslim agents. The last one indicates how the model is parameterized and how terrorist attack is designed in order to expose our 120 virtual populations of agents to the terrorist attack.

### Model

#### Overview

The model is based on the idea of cultural worldview given by [1] and inspired by Social Judgement Theory (SJT) [17]. We now propose an overview of the concepts that we use and their translation into the model.

- *Cultural worldview*. We assume that *K* cultural worldviews are available in the environment. A cultural worldview is a consistent set of concepts, beliefs, traditions, or rituals organizing the world and agent behavior. For instance, we consider Christian, Muslim, and areligious worldviews.
- *Positions about a cultural worldview*. Positions about a cultural worldview, indicating how much someone likes/adheres or dislikes/rejects it, are defined on a continuous axis from -1 (very negative) to +1 (very positive).
- *Agent acceptance segment about a cultural worldview*. Each agent has an acceptance segment on a cultural worldview. It is defined by its acceptable positions about this worldview, including its *most acceptable position*. The positions located in this segment are assumed acceptable for the agent whereas those outside of it are not acceptable. The most acceptable position, written *m*.*a*.*position* in the following, expresses the agent personal or preferred position about the worldview. It can be related to the most acceptable position of the SJT. Note that an agent may have positive acceptable positions about several worldviews. For instance, an agent can have high positive positions for the areligious worldview as well as lower positive positions for Christian or Muslim worldviews.
- *Lower and higher margin of acceptance for a worldview*. The acceptance segment is divided into a lower and higher margin of acceptance located around the m.a.position. The lower margin of acceptance is a segment of the [–1,1] axis, which is lower than the agent’s m.a.position; meanwhile, the higher margin of acceptance is a segment defined as similarly higher than the agent’s m.a.position. For convenience, the lower margin of acceptance is called *margin(l)* in the following discussion whereas the higher margin of acceptance is called *margin(h)*.
- *Cultural identity of an agent*. The segments of acceptance for the *K* available worldviews represent the cultural identity of an agent. They indeed express the agent’s acceptable positions about the different worldviews.
- *Attitude of an agent about an acceptance segment for a worldview*. We compute an agent’s attitude about an acceptance segment for a worldview by applying a similarity function between this segment and its own acceptance segment for this worldview. The result is positive if the segments strongly overlap (i.e. they share a large part of similar acceptable positions), and negative when they are separated and far apart. This is supported by SJT, which has shown that “the perceived distance depends on the level of involvement and the width of the latitude of acceptance” [18, 19].
- *Attitude of an agent about a cultural identity*. The attitude about a cultural identity is the average of the attitudes about the acceptance segments for the worldviews. To summarize, the more an agent perceives the cultural identity of another to be similar (i.e., in agreement to its own), the more its attitude about this agent is positive [6] whereas perceived differences lead to negativity. Indeed, “people exaggerate the value of those who share their worldview or who provide positive evaluations and denigrate the value of those with diverging worldviews or who provide negative evaluations” [20].
- *Cultural group of an agent*. We assume that, if asked to declare its membership in a cultural group (such as Christian, Muslim, areligious), the agent would answer the one corresponding to the worldview for which its m.a.position is the highest, which we also call the preferred worldview.

We use this model to simulate how a terrorist attack may change the cultural identities of the agents and, consequently, the attitudes of agents about each other (supposing that they are aware of all changes). We assume that, when perpetrating an attack, terrorists stress their cultural identity, such as extreme positive positions for the Muslim worldview and extreme negative positions for other worldviews, all forming very narrow acceptance segments. We assume that the agents whose cultural identity is perceived negatively by the terrorists [21, 22] feel a threat toward their identity [1] and decrease their acceptance segments on the most supported worldview by the terrorist (i.e. the Islamic worldview), in order to differentiate themselves from the terrorists’ cultural identity. Moreover, we assume that these agents reduce their acceptance segments more strongly when they perceive the terrorists’ identity to be relatively close to their own (yet still far enough to be threatening) than when they are already very different. We next describe the model in more detail.

#### The cultural identities of agents

We consider a population of *N* agents, with each agent *i* having a cultural identity defined by its acceptance segments on each of the *K* cultural worldview present in our society. In the present studies, we consider *K* = 3, with the Muslim, the Christians and the areligious cultural worldviews (abbreviated respectively M, C and A in the following and written as *k* ∈ {*M*,*C*,*A*} in the equations). The acceptance segments on M, C and A are described by:

- *K* values between -1 and +1, corresponding to its m.a.position $a_{k}^{i}$ pro or anti on each *k* cultural worldview of the *K* cultural worldviews present in the population. In the simulated populations, for each agent, there is at least one worldview for which the agent has a positive position. An agent’s identity group is defined by the worldview for which it has the highest m.a.position.
- *2K* values between -1 and +1, corresponding to the higher and lower bounds $B_{k}^{i}$ and $b_{k}^{i}$, representing the minimum and the maximum acceptable positions of the agent *i* on the worldview *k*. The segment going from $a_{k}^{i}\;to\;B_{k}^{i}$ is called higher margin or margin(h). The segment going from $b_{k}^{i}$ to $a_{k}^{i}$ is called lower margin or margin(l).

Thus, an agent cultural identity is represented by 2*K* + *K* values. In the present study, since K = 3, 9 values are necessary.

#### Differences between cultural identities determine agents’ attitudes about each other

Attitudes of agents about each other are computed from the comparison of their cultural identities. Each agent perceives its environment through its cultural identity, and its attitude about another agent also depends on its perception of the other agent’s cultural identity. Its attitude about its own cultural identity is at the maximum value: 1. An agent’s attitude toward another agent’s cultural identity is an average of its attitudes about the perceived segments of acceptance composing the other agent’s cultural identity. Thus the computation of the attitude of an agent toward another can be decomposed in three types of calculations: (1) Calculation of the agent’s attitude about a position (held by itself or another agent) on a cultural worldview *k*; (2) Calculation of the agent’s attitude for an acceptance segment (held by itself or another agent) on a cultural worldview *k;* (3) Calculation of the agent’s attitude toward a cultural identity (its own or the one of another agent).

(1) Calculation of the agent’s attitude about a position on a cultural worldview k

Considering the worldview *k* of agent *i*, the m.a.position and margins of acceptance can be used to compute the attitude $\omega_{i}^{k}(p)$ about a position *p* on the worldview *k* as follows:

$$\begin{array}{l} \omega_{k}^{i}(p)=1,if\;p<B_{k}^{i}\;and\;p>b_{K}^{i}\\ \omega_{k}^{i}(p)=\frac{e^{y}-1}{e^{y}+1}with\;y=1+\frac{p-a_{k}^{i}}{a_{k}^{i}-b_{k}^{i}},\;if\;p\leq b_{k}^{i}\\ \omega_{k}^{i}(p)=\frac{e^{y}-1}{e^{y}+1}with\;y=1+\frac{p-a_{k}^{i}}{a_{k}^{i}-b_{k}^{i}},\;if\;p\leq b_{k}^{i} \end{array}$$ (1)

**Fig 1:**
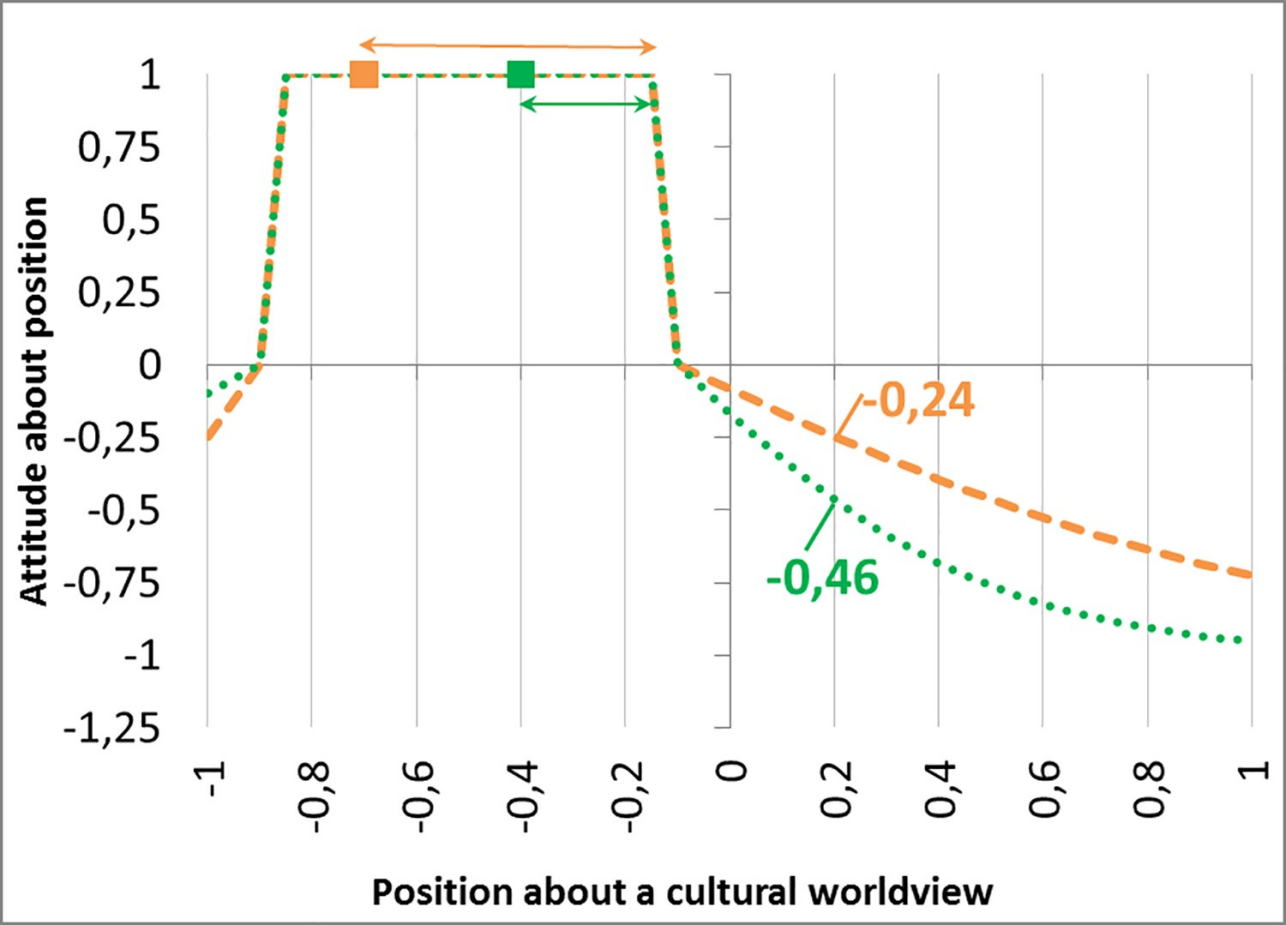
**Graphs of the attitude values (on the y-axis)** $\omega_{k}^{i}(p)$ **of agent *i* in orange, and** $\omega_{k}^{j}(p)$ **of agent *j* in green for possible positions *p* on a worldview (on the x-axis).** The values $\omega_{k}^{i}(p)$ depends on the acceptable positions of *i*. and the one of $\omega_{k}^{j}(p)$ depends on the acceptable positions of *j*. For both agents, the acceptable positions are recognizable in the figure as going from -0.85 to -0.15 on the x-axis since these positions are valued 1 on the y-axis Agents *i* and j have a similar acceptance segment but a different m.a.position (see the orange and the green squares respectively). Thus they have a different margin(h) (outlined by the orange and green arrows). The green agent, having a smaller margin(h) decreases more quickly the attitude given to positions outside its acceptance segment. A similar position 0.2 is valued -0.46 by the green agent while it is valued -0.24 by the orange agent.

We suppose that $\omega_{k}^{i}(p)$ equals +1 if *p* is in the margins of acceptance of *i*. If not, $\omega_{k}^{i}(p)$ is 0 for *p* at the bounds of the segment of acceptance and decreases in the distance between *p* and its closest bound of the segment of acceptance, with an asymptote at -1. Moreover, the decrease to -1 is faster for a smaller margin of acceptance. When the decrease is fast, the attitude of the agent can be almost the same (close to -1) for different values of attitude *p*. This function shape is illustrated in Fig 1.

(2) Calculation of the agent’s attitude for an acceptance segment on a cultural worldview k

To compute the attitude of an acceptance segment about another acceptance segment, the axis of positions for each worldview *k* is divided regularly into *D* values *p*_*d*_ (from -1 to +1). The attitude of *i* about the *j*’s acceptance segment on the worldview *k* is given by:

$$\omega_{k}^{ij}=\frac{\sum_{d=1}^{D}\omega_{k}^{i}(p_{d})max(\omega_{k}^{j}(p_{d}),0)}{\sum_{d=1}^{D}max(\omega_{k}^{j}(p_{d}),0)}$$ (2)

Note, that $\omega_{kk}^{ij}$ is maximal when the two acceptance segments are identical.

(3) Calculation of the agent’s attitude toward a cultural identity defined by the K acceptance segments

Finally, the overall attitude of *i* about *j* is the average attitude over the *K* different acceptance segments designing the agent’s total cultural identity:

$$\omega^{ij}=\frac{\sum_{k\in \{M,C,A\}}\omega_{k}^{ij}}{K}$$ (3)

Qualitatively, the agents with large margins of acceptance tend to have a positive attitude toward most of the others whereas agents with small margins of acceptance have very negative attitudes toward many others.

#### Impact of a threat on agent’s cultural identity

A terrorist attack is modeled by a scenario of messages in the media that convey the terrorists’ cultural identity. The acceptance segments of terrorists are assumed to be very small. On the Muslim worldview (abbreviated M), their acceptable positions are assumed to be very positive, while they are very negative on the others cultural worldviews

The agents of the population perceiving the terrorists’ attitude toward their cultural identity as negative are assumed to perceive a “threat” and modify their margins of acceptance. More precisely, let the cultural identities of terrorists *q* be defined by values $\left(a_{M}^{q},\;b_{M}^{q},B_{M}^{q}\right)$; the agents *i* then compute *ω*^*qi*^, the attitude of the terrorist *q* about them. If this attitude is positive, *i* is not scared and does not react. But if *ω*^*qi*^ is negative, *i* modifies its margins of acceptance away from the acceptance segments of *q* as follows.

If *ω*^*qi*^ < 0, the intensity of the margin of acceptance modification *μ* is:

$$\mu =\alpha \frac{e^{\omega^{qi}}-1}{e^{\omega^{qi}}+1}$$ (4)

where *α* is a positive number smaller than 1. The value of *μ* is close to -1 when *ω*^*qi*^ is very negative.

For *M* equal to the preferred worldview of the terrorist (i.e., the aggressor), the bound $\beta_{M}^{i}\in \{b_{M}^{i},B_{M}^{i}\}$ and which is the closest to $a_{M}^{q}$, is modified as follows (with *t* being the time):

$$\beta_{M}^{i}(t+1)=\beta_{M}^{i}(t)+\mu (\beta_{M}^{i}(t)-a_{M}^{i}-\varepsilon \frac{\left|\beta_{M}^{i}(t)-a_{M}^{i}\right|}{\beta_{M}^{i}(t)-a_{M}^{i}}))$$ (5)

where *ε* is a small positive number representing the smallest possible margin of the acceptance width (parameter of the model).

This change in the margins of acceptance results in a more negative attitude about the aggressor, in accordance with experimental observations [20].

### Generating virtual initial populations with cultural identities

We have seen in the previous subsection that an agent cultural identity is represented by 2*K* + *K* values. Then taking *K* = 3 worldviews (Christian, Muslim and Areligious), we need 9 values, chosen between -1 and +1, with constraints on their order, to define initially one agent. Taking two prototypical agents for each population group, we need 54 values to define the population of six agents. Then, even for such a small population of six agents, it is impossible to explore at random possible initializations of our agents.

This is why we concentrate our study on a particular case of attitudinal relationships between groups of agents supporting different worldviews. This particular case corresponds to attitudes of groups, towards other groups and themselves, defined by the analysis of survey data presented in the next subsection. From this data, we define a simple population of the 6 agents with different typical cultural identities. The second subsection presents this definition, as well as the optimization process leading to 120 initial populations that have attitudinal relationships between groups close to the ones from the data.

#### Attitudinal relationship between groups extracted from survey data

A survey on group’s attitudes in France has been used to initialize the model [16]. The survey questions are presented in S1 Appendix, in both the original languages, French, and English. An informed consent was filled by all participants. Data in text format (in S1 Data) and the code book (in S2 Appendix) are available in Supporting Information files. All data were fully anonymized prior to being accessed by the authors. The survey was conducted in accordance with the 1964 Helsinki declaration and was approved by an external Ethics Committee from the psychology department of the Université de Montréal, Canada. The age range of the participants was 18 to 89. The survey was conducted by an external agency as part of the research project “IMAG” funded by the National Research Agency in France.

**Fig 2:**
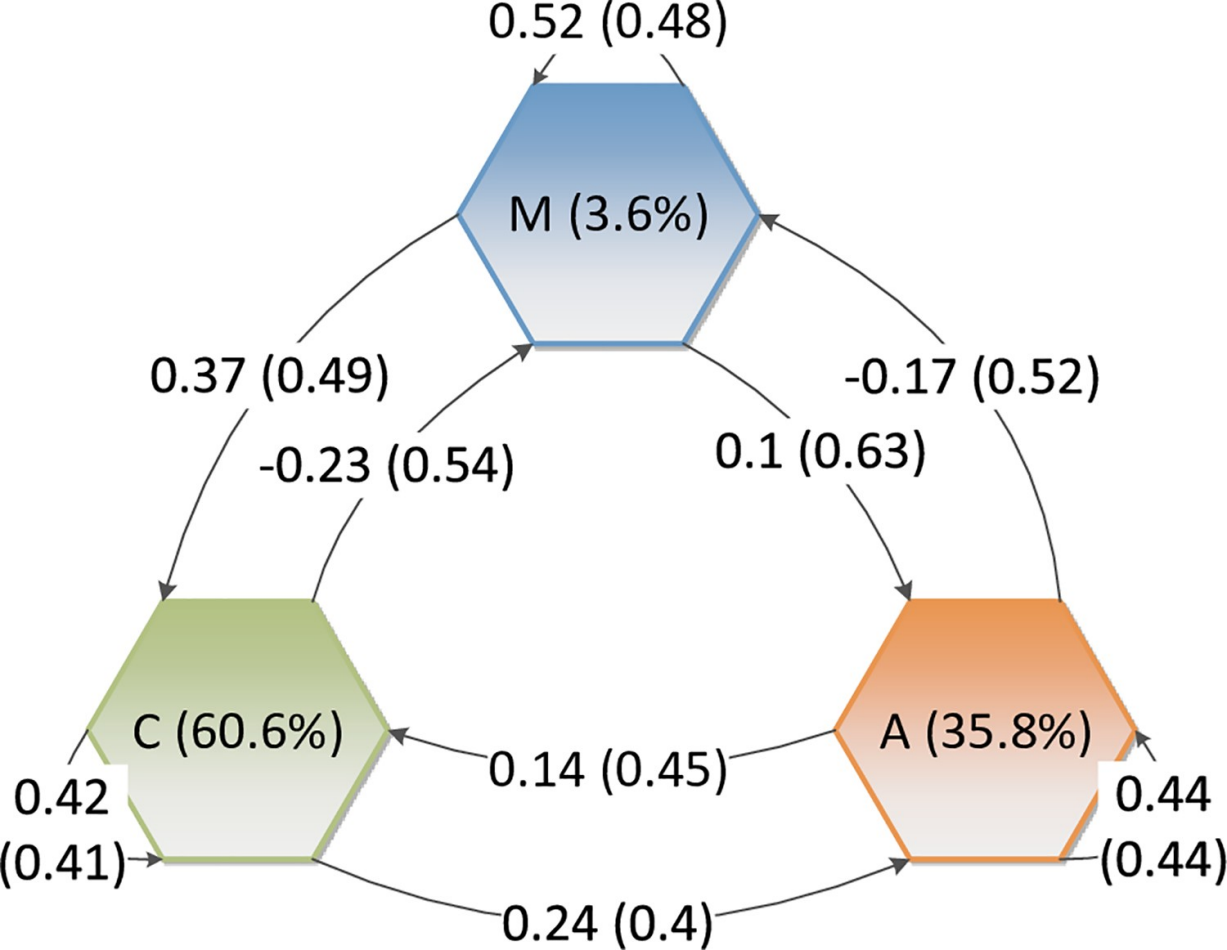
Attitudes of each group—Muslims (*M*), Christians (*C*), and areligious (*A*)—toward each other (averages and standard deviations in parentheses for each relation computed from the representative survey of the French population). Negative values represent the “unfavorable” attitudes. Positive values represent the “favorable” attitudes. The percentages in the middle of each hexagon is the percentage of people each group represents in the survey–it is close from their distribution in the French population: 3.6¨% for Muslims (*M*), 35.8% for areligious (*A*); 60.6% for Christians (*C*).

We wanted our virtual groups (defined by their preferred worldview) composing our populations to respect the measured attitude of the groups about themselves (in-group attitudes) and about other groups (intergroup attitudes). The surveyed sample includes 1000 people, representative of the French population. People answered to the question « what is your general attitude about the following groups?». The groups are A (for Areligious), M (for Muslims), and C (for Christians). They have to answer using a five-point scale ranging from “strongly unfavorable” to “strongly favorable”. Averages and standard-deviations for in-group attitudes and every out-group attitudes have been computed and normalized between -1 to +1 for modeling purpose. Fig 2 shows the groups’ attitudes.

#### Building virtual initial populations

The cultural identity of our population of virtual agents was defined by their acceptance segments for three cultural worldviews: *C*, *M*, and *A*. An agent’s preferred worldview defines the agent’s cultural group. We have seen that an agent’s cultural identity is represented by 9 values. Thus this is not possible to initialize differently a population of 1000 agents requiring defining 9000 values, with particular constraints of order between some of them. Then we decide to represent the diversity of the population with 6 typical cultural identities, depending at first of group of the agent, and secondly if an agent is inclusive or exclusive. Indeed, we assume that each group includes two types of agents:

- *Agents with exclusive identities*: these agents have most of their acceptance segment in the positive side for the worldview *k* ∈ {*M*,*C*,*A*} defining their group, and on the negative side for the other worldviews. Amongst these agents, we identify the extremists with the most acceptable positions close to 1 or -1 and narrow acceptance segments.
- *Agents with inclusive identities*: these agents have one or two positive m.a.positions and corresponding acceptance segments on the positive side of the position axis, as well as one or two others close to zero, with wide acceptance segments also mostly on the positive side.

Thus, in each group (*C*, *M*, or *A*) of the initial population includes *x*% of inclusive and *y*% of exclusive agents with their highest m.a.position for the worldview defining their group.

Overall, such a simple definition of the population is modelled by 57 values which cannot be chosen at random since “being a member of one particular group”, and “having inclusive or exclusive identities” requires particular relationships between the acceptance segments. Even with this simple definition population, building a population of agents remains a challenge since choices have to be done in a space of 57 dimensions.

We built at first typical cultural identity for each of our 6 types of agents. From there, we built collections of groups of *C*, *M* and *A* agents respecting each, the attitudes each group has toward itself in the survey presented earlier.

We compose populations from these groups, varying the groups, as well as the part of inclusive and exclusive agents in each group. We compute, for each population, the attitudes groups have toward each other. The quality of a population is defined by the sum of the absolute differences of the average attitudes and the standard deviations of each group toward the other and itself computed on the virtual population and on the data. We kept the 20 populations having the lowest errors.

These 20 populations are used to initialize a Particle Swarm Optimization algorithm in order to improve the population by minimizing the error. Indeed, Particle Swarm Optimization method [23] starts with a set of populations of possible solutions (which can be initialized following various techniques), and then makes iterative modifications of the populations, exploring at random around the current values with a tendency to explore closer to the best populations.

The 120 best populations obtained as results of this stochastic optimization algorithm are selected for our study of the evolution of the attitude toward the group of agents *M* after facing a terrorist attack. They have relative average errors of average attitudes and standard deviations ranging from 5% to 7%, and maximum errors ranging from 21% to 44%.

**Parameterizing the evolution of the population**

Once we have virtual populations, we want to simulate their evolution facing a “threat” message conveying the cultural identity of a terrorist.

The terrorists have very narrow acceptance segments with a very positive m.a.position for the worldview *M* defining their group and very negative m.a.positions about the two other worldviews (*C* and *A*). Thus, they tend to have a very negative attitude about all other cultural identities.

The parameters of the dynamics take the following values: *α* = 0.5, *ε* = 0.05, and *D* = 400.We simulate the evolution of our 120 populations facing “threat” messages. Due to the high level of media coverage and related discussions after a terrorist attack, agents are considered to be perfectly informed about the others’ acceptance segments after the attack. We investigate the result to identify how the population’s average attitude toward the cultural identities of the inclusive and exclusive agents of group *M* evolves (excluded the attitudes for terrorists).

## Results

### General results for the 120 populations

As the impact of the agent differentiation process from the *M* terrorist’s extreme cultural identity is to decrease the agent’s attitude toward the terrorist *M*, we expect that on average, agents of our populations decrease as well their attitude toward the group of *M* agents (excluding the terrorist agent). This is the case in most of our populations: the average attitude toward group *M*’s cultural identities decreases. However, it increases for some. The challenge is thus to understand why and when a population becomes more positive toward *M* non-terrorist agents, after facing a threat from terrorist *M* agents.

**Fig 3:**
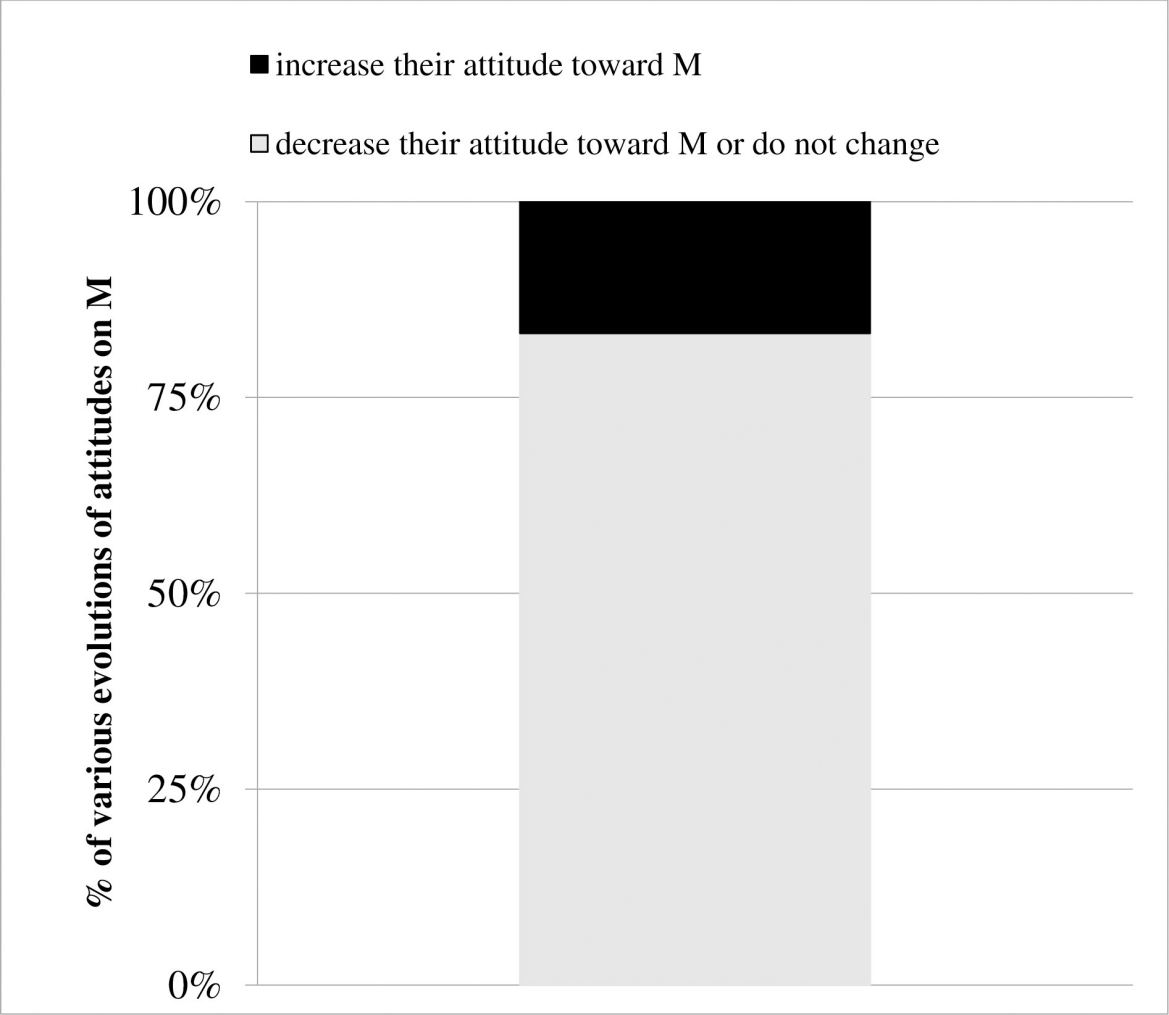
Distribution in percentages of types of evolutions of agent’s attitude toward *M* agents (increase in black, decrease or do not change in grey) for all initial 120 populations and times.

We compute the evolutions of attitude toward *M* agents of every agent composing our populations: some agents increase their attitudes toward *M* agents, while other agents decrease or do not change their attitudes toward *M* agents. Fig 3 shows the percentage of each evolution (increase or decrease attitude about *M*) for the agents of our 120 populations. It confirms that a majority of agents decreases or do not change their attitude toward *M* agents (83%, in grey). However, a minority of agents increases their attitude toward *M* agents (17%, in black).

### Relationships between groups leading to an increase of their attitude to agents of group *M*

We then study the agents in populations increasing their attitudes about group *M*, compared to populations decreasing their attitudes about group *M*. We identify the agents’ cultural identity properties which explain one or the other evolutions of a population attitude toward group *M*.

#### Reactions of agents when they are exposed to a threat

During the simulations, agents change their margin(h) (part of the acceptance segment located higher than the m.a.position) on the *M* worldview. In populations increasing their average attitude about group *M*, we observe the following particular reactions to terrorist attacks:

- *M* agents strongly decrease their higher margin of acceptance (margin(h)) of the *M* worldview; and
- Non-*M* (especially *C*) agents almost do not decrease their margin(h) of the *M* worldview.
  Such reactions are explained by particular properties of the cultural identities of agents in these populations, compared to the properties of the cultural identities of agents in the other populations:
- *M* agents: the margin(h) of acceptance are larger, allowing a strong decrease.
- Non-*M* agents: the margin(h) of acceptance is very small, implying almost no change in its width

#### Evolution of attitudes of two agents toward each other when they are exposed to a threat

To have a clear overview of the properties of the cultural identities of *M* and non-*M* agents interactions leading to an increase of the attitude of the non-*M* agents toward the *M* agent, we study analytically the change of attitude on the *M* cultural worldview. Indeed, since the *M* acceptance segment is the only acceptance segment possibly changing due to the terrorist attack, the study of the impact of this change of the attitude of one self’s cultural worldview on another agent’s acceptance segment on the same worldview, is sufficient to conclude about the direction of the global change of attitude of an agent toward another.

Then, let’s consider two agents *i* and *j* and their possible change of attitude toward each other due to the change of their segment of acceptance on worldview *M* when one of the two bounds of the agents, $B_{M}^{i}$ and $B_{M}^{j}$, possibly decreases due to the impact of a cultural threat of a terrorist agent *q* on *M*. The evolutions are called ${\Delta B}_{M}^{i}$, with ${\Delta B}_{M}^{i}=B_{M}^{i}(t^{1})-B_{M}^{i}(t^{0})$, and similarly for *j, ${\Delta B}_{M}^{j}$.* We assume $a_{M}^{i}\leq a_{M}^{j}<a_{M}^{q}$. The agent *i*’s attitude about *j* is sensitive to a possible overlap between its acceptance segment and *j*’s acceptance segment, (i.e. part of their acceptance segments corresponding to common acceptable positions). The overlap *o* measures the similarity between *i’*s and *j’*s views. It is valued (max($b_{K}^{i};b_{K}^{j}$)–min($B_{K}^{i};B_{K}^{j}$)). When it is positive, it indicates the level of similarity. When it is negative, it indicates the level of dissimilarity. Then, for each studied case in the following, we give details on the value of the change of attitude of *j* when *j* initially perceives *i* as very different (*o* < 0), and when *j* initially perceived *i* as partly or totally similar (*o > 0*). We illustrate some interesting point by figures. We draw up the attitudinal values of the positions on the worldview *M* of two agents: a non-*M* agent (represented in orange by a plain line), and a *M* agent (represented in blue by a plain line). The positions valued 1 by one or the other agent correspond to the positions defining their acceptance segments. The dotted line represents the states of the two agents before the aggression; the plain line shows the values after the aggression.

We start with the agent *j*, with the higher m.a.position, $a_{M}^{j}$, and its change of attitude toward *i* when the margin(h) of *i* and *j* possibly decrease. In this case, *j* is the non-*M* agent, and *i* is the *M* agent. Since we assume $a_{M}^{i}\leq a_{M}^{j}$, we show the change in the *j*’s attitude for the *i*’s acceptance segment on the cultural worldview *M, ${\Delta \omega }_{M}^{ji}$*, is always lower or equal to zero, ${\Delta \omega }_{M}^{ji}\leq 0$.

When *j* initially perceives *i* as different (*o* < 0), since *j* values *i* through its margin(l) which is not changed by the terrorist attack, we have ${\Delta \omega }_{M}^{ji}=0$ if ${\Delta B}_{M}^{i}=0$, or we have ${\Delta \omega }_{M}^{ji}∼0$ if ${\Delta B}_{M}^{i}<0$. Indeed, if we consider *i* for example, if $B_{M}^{i}(t^{0})$ et $B_{M}^{i}(t^{1})$ are very far from $a_{M}^{j}$ and/or the margin(l) of *j* is small, then we have ${\Delta \omega }_{M}^{ji}∼0$ whatever ${\Delta B}_{M}^{i}$ and ${\Delta B}_{M}^{j}$ due to the form of the “perception” equation (1 b or c) which implies everything far enough is equally perceived as far. When *j* initially perceives *i* as partly or totally similar (*o > 0*), we have ${\Delta \omega }_{M}^{ji}<0$ if ${\Delta B}_{M}^{i}<0$ whatever ${\Delta B}_{M}^{j}$ since: (1) the acceptable positions of *i* valued on the base of the margin(h) of *j* becomes always more negative when ${\Delta B}_{M}^{j}<0$; (2) the part of acceptable positions of *i* valued by *j* though the lens of its margin(l) ${(a}_{K}^{j}-b_{K}^{j}),$ keeps the same value if it remains the same after the threat, or decreases in value if it decreases in width after the threat.

To sum-up, if the non-*M* agent has a higher m.a.position for worldview *M* than the *M* agent, the non-*M* agent, whatever its change and the change of the *M* agent, does not change, or decrease, its attitude toward the *M*-agent. This is true since a change in an acceptance segment always remains stable, or decreases the non-*M* perceived similarity with the *M* agent.

We continue our systematic study with the agent *i*, having the lower m.a.position, $a_{M}^{i}$, compared to the m.a.positions of the agent *j* and the terrorist *q*. We study the change of attitude of *i* toward *j* when the margins(h) of *i* and *j* possibly decrease. In this case, *j* is the *M* agent, and *i* is the non-*M* agent.

For simplicity sake, we assume, as noticed from the observed evolutions of the agents in the simulated populations leading to an increase of the attitude toward *M* agents, that one agent, the non-*M* agent, does not change its acceptance segment, ${\Delta B}_{M}^{i}=0$, while the other, the *M* agent, decreases its acceptance segment, especially decreases its margin(h) on the *M* worldview. The assumption that the non-*M* agent does not–or almost does not- change its acceptance segment implies that: (1) the attitude of the terrorist about the non-*M* agent is positive; or (2) the margin(h) of the non-*M* agent is very small and cannot decrease its width much. By hypothesis over the terrorist’s cultural identity, the condition 1 is not possible. Then we have to retain that we have thus a non-*M* agent with a very small margin(h).

**Fig 4:**
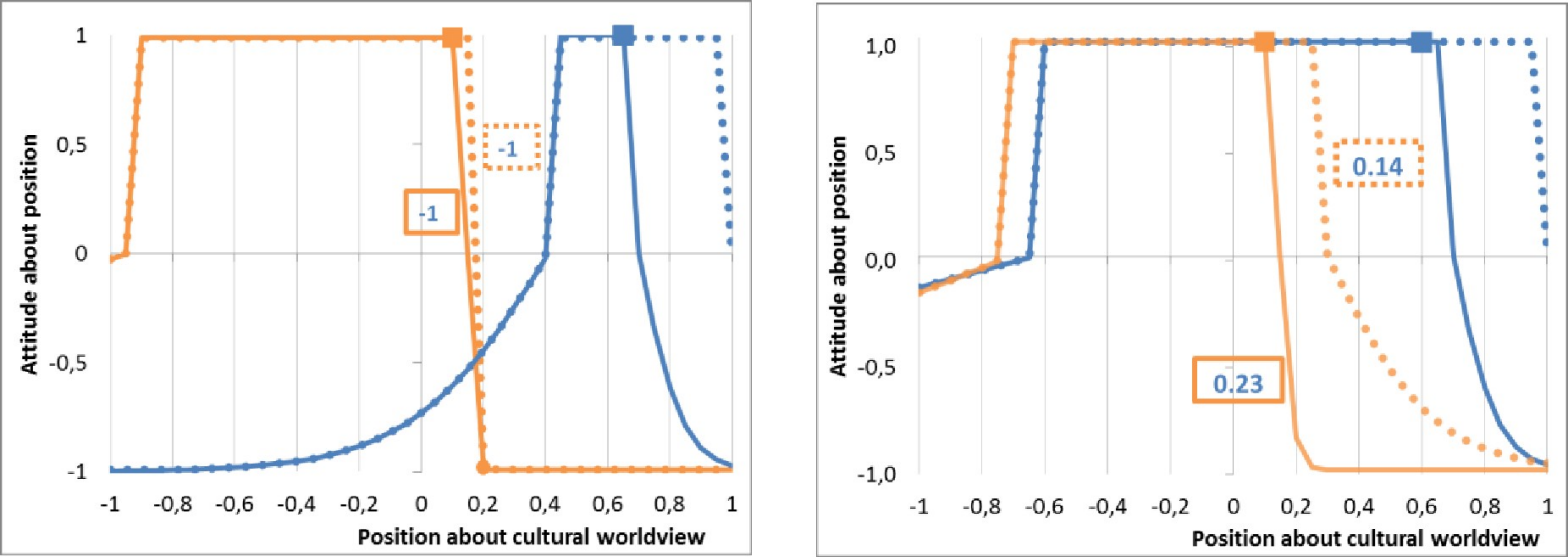
Evolution of the attitude of non-M agent (in orange) for the M agent (in blue): Dotted line, before the terrorist attack; plain line, after the agression. (a) On the left, the attitude does not change despite the change of M margin(h) of acceptance, it remains -1: the maximum change of M margin(h) is limited by $a_{M}^{j}$ which is valued -1 by i in any cases. Thus the attitude of the non-M agent for the M agent can’t change; (b) On the right, the attitude decreases (from 0.23 to 0.14) due to the decreasing of the margin(h) of acceptance of the non-*M* agent and despite the part of the overlap over the *M* acceptance segment has increased. Indeed, with a smaller margin(h), the non-*M* agent sees the part of the inclusive *M*’s segment external to it strongly more negatively than before.

When *i* and *j* are totally dissimilar (*o* ≤ 0), since the change in *i*’s perception of *j*’s closeness is valued through the lens of its not assumed constant, or almost constant, very small margin(h) ${(B}_{M}^{i}-a_{M}^{i})$: we have ${\Delta \omega }_{K}^{ji}=0$ since the m.a.position of *j* is valued -1 by *i* (which is quasi almost true for a very small margin(h) of *i)*. Indeed, in such a case, whatever the negative value of ${\Delta B}_{M}^{j}$, *j* always perceives *i* as very different. This case is illustrated by the Fig 4A. We can here easily infer that a ${\Delta B}_{M}^{i}<0$, the result is the same: ${\Delta \omega }_{K}^{ji}=0$.

**Fig 5:**
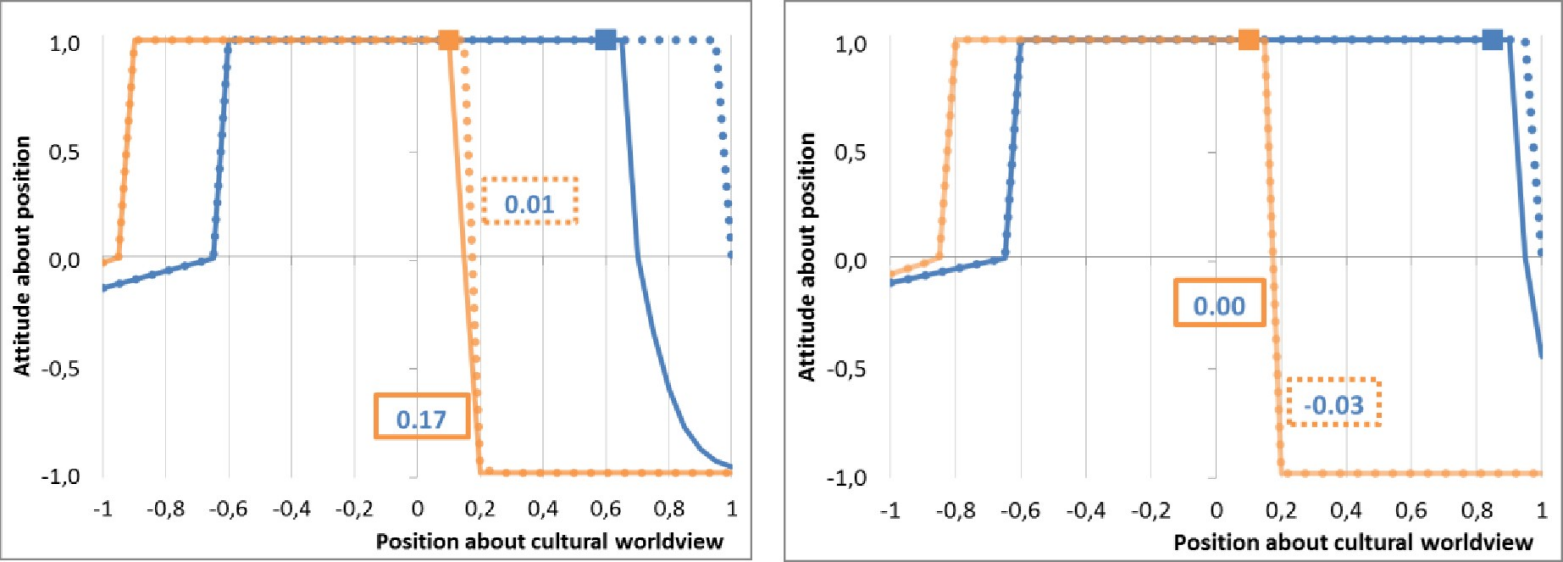
Evolution of the attitude of the non-M agent about the M agent (in blue): Dotted line, before the terrorist attack; plain line, after the aggression. (a) On the left, the attitude increases (from 0.01 to 0.17) due to the very small decreasing of the margin(h) of acceptance of the non-*M* agent, and the part of the overlap over the M acceptance segment has increased; (b) On the right, the attitude increases (from -0.03 to 0.00) due to the very small decreasing of the margin(h) of acceptance of the non-M agent, and the part of the overlap over the M acceptance segment has increased.

When *i* is and *j* are partly or totally similar (*o* > 0), we have ${\Delta \omega }_{M}^{ij}>0$ when *i* perceives *j* as close, at least after *j* has rejected the extremists, through the lens of its not changing margin(h) ${(B}_{M}^{i}-a_{M}^{i})$. This is illustrated by the Fig 5A. If *i* perceives *j* as very different before and after the threat, due to its small margin(h) defining the perception of the difference and a slightly negative ${\Delta B}_{M}^{j}$ (i.e. a small rejection of terrorist’s identity but the *M* agent), we can also have ${\Delta \omega }_{M}^{ij}>0$ when the part of *j*’s segment that the overlap represents, increases sufficiently (see the Fig 5B for an example). Globally, the reaction function to the terrorist’s attack tends to imply that the larger the *j*’s margin(h), the larger the ${\Delta B}_{M}^{j}$. Then we can conclude that the larger the *j*’s margin(h), the larger the ${\Delta B}_{M}^{j}$, and the larger ${\Delta \omega }_{M}^{ij}.$ In other words, the increase of attitude of *i* toward *j* is larger when the *j*’s margin(h) is larger. On the other hand, ${\Delta B}_{M}^{j}$ is bounded by $a_{M}^{j}$, which means for a constant $B_{M}^{j}$, the lower $a_{M}^{j}$, the larger the ${\Delta B}_{M}^{j}$, and the larger ${\Delta \omega }_{M}^{ij}$. Since we have assumed $a_{M}^{i}\leq a_{M}^{j}<a_{M}^{q}$, this means that closer are $a_{M}^{j}$ and $a_{M}^{i}$, and the larger ${\Delta \omega }_{M}^{ij}$.

In the other cases, as for ${\Delta B}_{M}^{j}=0$, we have ${\Delta \omega }_{M}^{ij}=0$. In order to discuss the fact that we have assumed, starting from the observed simulated population, that the non-M agent almost do not change its margin(h), Fig 4B illustrates what occurs when there is not a very small margin(h) of acceptance of non-*M* (orange) agents. Despite the increasing overlap in the *M* acceptance segment, the decreasing margin(h) of acceptance of the non-*M* is such that it indicates the *M* as being very much further than before. When there isn’t a very small margin(h) of acceptance of non-M agents, non M-agents are not indifferent to the threat. Then, despite the increasing part of the overlap of the segment due to large margins(l), the decreasing of the margin(h) of acceptance of the non-*M* agent is such as it perceives the *M* agent as very much further than before (for example the position 0.4 is value -1 (after on the right), while it was previously valued -0.28).

Overall, a non-*M* agent increases its attitude over a *M* agent after a terrorist attack if *M* and non-*M* agents’ cultural identities show altogether the following properties:

1. the m.a.position of the non-*M* agent is lower than the m.a. position of the *M* agent on the *M* worldview;
2. the non-*M* agent has a small margin(h) which almost does not decrease in width after the terrorist attack;
3. *M* and non-*M* agents have common acceptable positions which should remain after the terrorist attack. These acceptable positions are, at least partly based on overlap of the margins(l) of the *M* and the non-*M* agents since these margins are not changed by the terrorist attack. Thus, the larger the margins(l) of the *M* and non-*M* agents, the higher the probability to observe an increase of attitudes over *M* agents;
4. the increase of the non-*M* agent attitude toward the *M* agent attitude is higher when the *M* agent’s margin(h) is larger, and when the distance between the m.a.positions of the non-*M* and *M* agents is smaller; in other words, this means *M* agents should differentiate strongly from the terrorist’s cultural identity and that non-*M* agent should be supportive of the *M* worldview (i.e. the m.a.position of the non-M agent is positive, even if it is lower than the one of the *M* agent).

The analysis of the specific properties of the cultural identities of *M* and non-*M* agents in the simulated populations having a final increase of the average attitude toward the *M* agents confirms the result of our analytical study.

Let us reformulate in other words what the necessary cultural identities’ properties means for a population that will increase its average attitude toward *M* agents after a terrorist *M* attack:

1. *M* agents should be the most supportive of the *M* worldview;
2. non-*M* agents do not change their cultural identity after the terrorist attack;
3. *M* and non-*M* agents should have common acceptable positions on the *M* worldview that remain common after the attack;
4. *M* agents should strongly differentiate from the terrorist cultural identity, and the non-*M* agents should have a positive m.a.position on *M*, even if it is lower than the m.a.position of the *M* agents, and their margin of acceptance above their m.a.position should be small.

## Discussion and conclusion

The modeled populations increasing their attitude about the group of main worldview *M* to which a terrorist group is assimilated, show specific initial acceptance segments about this worldview. These specific acceptance segments represent particular cultural identities which should be sufficiently present in a population to observe an average increase of the attitude toward *M* agents of the population after a terrorist attack. They imply:

1. *M* agents are the most supportive of the *M* worldview, but non *M* are also quite supportive, even if intolerant toward extremist *M* positions;
2. non-*M* agents do not change much their cultural identity after the terrorist attack since they consider as not acceptable the positions slightly higher than the positions they accept the most on the *M* worldview;
3. *M* and non-*M* agents should have a large set of common acceptable positions on the *M* worldview;
4. *M* agents strongly differentiate from the terrorist cultural identity after the attack,.

These specificities permit to non-terrorists *M* agents to strongly differentiate from terrorist’s cultural identity, and to other agents to perceive this change, and to value it positively.

This work outlines that it is not only the part of *M* agents in the population which defines the type of reaction a population has toward them after an attack of external agents supporting the *M* worldview. Indeed, this is defined by the distribution of particular cultural identities, not only of *M* agents, but also of non-M agents in the population. To ensure that the hostility about M does not increase after a terrorist attack, it is fundamental there are non-*M* agents having a positive m.a.position about the *M* worldview, even if it is not their preferred worldview in the population. It is also of importance that, both *M* and non-*M* agents, have a large tolerance for positions that are lower than their m.a.position (included “against” positions).

The importance of a large part of common accepted positions about the Muslim worldview can be related to the feeling of similarity and solidarity observed in the massive French demonstrations that took place after the Charlie Hebdo terrorist attacks. They can also be related to the results of [6, 7]. Indeed, we observe that some cultural properties, when simultaneously becoming salient with the threat, may increase perceived similarity of members from different groups and, thus, avoid an increase in intergroup hostility.

It should be underlined that, in the model, the decrease of hostility about Muslims takes place because the agents of the Muslim group reject strongly the radical cultural identity of the terrorists, and that the other agents are aware of their evolution.

The other important point is that the non-*M* agents do not become more negative toward terrorist agents. This can be related to the famous “you won’t have my hatred” from (Antoine Leiris, Facebook, 16th of November 2015) which has been strongly diffused and positively discussed by French people. It can also be related to the “not afraid”, that has been published and asserted by many people after the Charlie’s terrorist attack, to indicate the willingness to resist any change because of the attacks.

Overall, the results of this simulation are quite consistent with recent research related to TMT. They suggest that cultural worldviews allow agents to cope with important repeated threats and that becoming negative toward those who have a different cultural worldview is only one of many possible reactions.

The next step is a deeper study of the link between the cultural identities of groups’ members and some particular attitudinal relationship between groups. We can notice for example that in the French surveyed populations, Christians and Muslims have more asymmetrical relationships than Areligious and Muslims. Another interesting question is about the various evolutions of attitudes, not only toward Muslims on average, but also of each group toward itself and other groups.

Further, we expect to design and perform experiments to check and improve the model.

## Supporting information

S1 Appendix “Original survey script”. (PDF)

S2 Appendix “Code book of the survey”. (PDF)

S1 Data This is a text file which can be opened as a table, or a csv file. The variables are: identifier of the participant, region of France, age, gender, attitude toward Christians, attitude toward Muslims, attitude toward areligious, religion, religion (others), time of beginning of the survey, time of end of the survey.
(TXT)
