# Supplementary material for: Resisting hostility generated by terror: An agent-based study
Source: PLoS One. 2019 Jan 14;14(1):e0209907. doi: 10.1371/journal.pone.0209907 (PMC6331148; doi:10.1371/journal.pone.0209907)
Supplement: S1 Appendix — (PDF) [file pone.0209907.s001.pdf]

Original Survey Script – Translation in English in red

Dans quelle région habitez-vous? In which region do you live ?

<1> Nord-Est
<2> Nord-Ouest
<3> Region Parisienne
<4> Sud-Est
<5> Sub-Ouest

Quel est votre âge? How old are you ?

Quel est votre sexe? What is your gender ?

<1> Femme - Female
<2> Homme - Male

Quel est le niveau de diplôme le plus élevé que vous avez atteint? Which is your highest qualification? (in the following various degrees of diploma, from no to the highest)

<1> Sans diplôme
<2> Non diplômé(e) jusqu'à la fin de la 2ème, 2nde, 1ère filière générale
<3> Non diplômé(e) du CAP BEP filière professionnelle
<4> Certificat d'études primaires
<5> CAP, examen de fin d'apprentissage artisanal
<6> BEP, BP, BEA, BEC, BEI, BES
<7> Brevet élémentaire, Brevet d'étude du premier cycle, brevet des collèges
<8> Baccalauréat général, Brevet supérieur
<9> Brevet de technicien, baccalauréat de technicien, baccalauréat technologique, baccalauréat professionnel
<10> Diplôme universitaire de premier cycle (DEUG), diplôme universitaire de technologie (DUT), Brevet de technicien supérieur (BTS), Certificat d'Aptitude Pédagogique
<11> Diplôme universitaire des deuxième et troisième cycles, Doctorat, CAPES, Agrégation, Diplôme de grandes écoles

Opinions, politiques publiques et représentations sociales

Opinions, public policy and social representations

Roxane de la Sablonnière, Université de Montréal
Serge Guimond, Université Blaise Pascal

A. RENSEIGNEMENTS AUX PARTICIPANTS – INFORMATION TO PARTICIPANTS

Objectifs de la recherche – Purpose of the research

Ce projet de recherche vise à étudier l'opinion publique concernant les questions d'actualité, les normes culturelles et les représentations sociales.
This research project aims to study public opinion related to social issue, cultural norms and social representations.

Participation à la recherche – Participation to the research

Votre participation à cette recherche consiste à remplir un questionnaire composé de mesures portant sur les normes culturelles et diverses attitudes.
Your participation to this research consists in filling a questionnaire composed of measures on cultural norms and various attitudes.

La participation à la recherche requiert environ 20 minutes. The participation to the research takes about 20 minutes.

Confidentialité - Confidentiality

Il s'agit d'une étude anonyme. Personne ne pourra vous identifier. Aucune information permettant de vous identifier d'une façon ou d'une autre ne sera publiée.
This is an anonymous study. Nobody will be able to identify you. No information allowing identifying you on one way or another will be published.

Avantages et inconvénients – Strengths and weaknesses

En participant à cette recherche, vous pourrez contribuer à l'avancement des connaissances en psychologie sociale. De plus, votre participation pourra également vous donner l'occasion de mieux vous connaître personnellement. Par contre, il est possible que votre participation à cette étude suscite des réflexions ou des souvenirs émouvants ou désagréables. Si cela se produit, n'hésitez pas à contacter l'agent(e) de recherche. S'il y a lieu, l'agent(e) de recherche pourra vous référer à une personne-ressource.
By participating in this research, you will be able to contribute to the advancement of knowledge in social psychology. In addition, your participation may also give you the opportunity to get to know you better personally. On the other hand, it is possible that your participation in this study provokes reflections or memories moving or unpleasant. If this happens, do not hesitate to contact the research officer. If applicable, the Research Officer may refer you to a resource person.

Droit de retrait – Withdrawal rights

Votre participation est entièrement volontaire. Vous êtes libre de vous retirer en tout temps, sans préjudice et sans devoir justifier votre décision.

Your participation is entirely voluntary. You are free to withdraw at any time, without prejudice and without justifying your decision.

B. CONSENTEMENT - CONSENT

En répondant au questionnaire, vous indiquez par le fait même que vous acceptez de participer à cette recherche. Pour toute question relative à la recherche, ou pour vous retirer de la recherche, vous pouvez communiquer avec Roxane de la Sablonnière (professeure agrégée au département de psychologie de l'Université de Montréal)au numéro : 001-514-343-6111 pound.raw 6732 ou à l'adresse courriel roxane.de.la.sablonnière@umontreal.ca ou avec Serge Guimond (professeur des Universités, 1ère classe, UFR psychologie de l'Université Blaise Pascal) au numéro suivant : 04.73.40.62.51 ou à l'adresse courriel serge.guimond@univ-bpclermont.fr. Toute plainte relative à votre participation à cette recherche peut être adressée à l'ombudsman de l'Université de Montréal, par téléphone 001-514-343-2100 ou à l'adresse courriel ombudsman@umontreal.ca. Notez que l'ombudsman de l'Université de Montréal accepte les appels à frais virés.
By answering the questionnaire, you indicate by the fact that you agree to participate in this research. For any questions relating to the research, or to withdraw from the research, you can contact Roxane de la Sablonnière (Associate Professor in the Department of Psychology at the Université de Montréal), at the number: 001-514-343-6111 pound.raw 6732 or at the email address: roxane.de.la.sablonniére@umontreal.ca or with Serge Guimond (University Professor, 1st class, UFR Psychology, Blaise Pascal University) at the number: 04.73. 40.62.51 or the email address: serge.guimond@univ-bpclermont.fr. Any complaint relating to your participation in this research may be addressed to the ombudsman at the Université de Montréal, by phone 001-514-343-2100 or at the following email address: ombudsman@umontreal.ca. Note that the ombudsman at the Université de Montréal accepts collect calls.

C. QUESTIONNAIRE - QUESTIONNAIRE

Nous avons tous des réactions plutôt positives ou plutôt négatives face à différentes personnes ou groupes de personnes. En utilisant l'échelle ci-dessous, veuillez indiquer quelle est votre attitude générale envers les groupes suivants.
We all have rather positive or rather negative reactions to different people or groups of people. Using the scale below, please indicate your general attitude towards the following groups.

- Les Chrétiens : Christians
- Les Musulmans : Muslims
- Les athées : Areligious

Echelle : Scale
<1> Très défavorable – Very unfavourable
<2> Défavorable - Unfavourable
<3> Neutre - Neutral
<4> Favorable - Favorable
<5> Très favorable – Very Favorable

Veuillez indiquer quelle est votre religion ou votre confession si vous en avez une :
Please indicate what is your religion or confession if you have one:

<1> Catholique Catholic
<2> Protestante Protestant
<3> Musulmane Muslim

<4> Juive Jewish
<5> Hindouiste, Bouddhiste Hinduist, Buddhist
<6> Agnostique Agnostic
<7> Athée Atheist
<8> Autre. Other.
