# Supplementary material for: Resisting hostility generated by terror: An agent-based study
Source: PLoS One. 2019 Jan 14;14(1):e0209907. doi: 10.1371/journal.pone.0209907 (PMC6331148; doi:10.1371/journal.pone.0209907)
Supplement: S2 Appendix — (PDF) [file pone.0209907.s002.pdf]

==
========
Project Code: LAPS0001
Project Name: Attitudes toward Immigrants in France
Prepared for: Emmanuelle Kleinlogel (Serge Guimond)
Interviews: 1000
Field Period: December 22, 2014 - December 31, 2014
Project Manager: Ashley Grosse - 650.462.8016
==
========
YouGov interviewed 1057 respondents who were then matched down to a
sample of 1000 to produce the final dataset. The respondents were
matched to a sampling frame on gender, age, and residence in one of
five larger French regions. The frame was constructed based on
population
marginals provided by Insee (Institut national de la statistique et
des
études économiques) The matched cases were weighted to the marginals
of
the sampling frame using iterative proportional fitting (IPF). The
targets
were the joint distribution of five category age by gender, and the
marginal distribution for five category region (Nord-Est, Nord-Ouest,
Region Parisienne, Sud-Est, Sud-Ouest) and four category eduction.

Variable List
==

Name Description
---- -----------
caseid Case ID
region Region
block20_age Age
block20_gender Gender

block16_9 Ingroup bias and outgroup
evaluation - block16_9
block16_10 Ingroup bias and outgroup
evaluation - block16_10
block16_11 Ingroup bias and outgroup
evaluation - block16_11

block21_religion Religion
block21_religion_t Religion – Open

Variable Map and Codebook
==
========
Name: caseid
Description: Case ID
==
========
Name: region
Description: Region
Count Code Label
----- ---- -----
185 1 Nord-Est
246 2 Nord-Ouest
213 3 Region Parisienne
231 4 Sud-Est
125 5 Sub-Ouest
0 8 Skipped
0 9 Not Asked
==
========
Name: block20_age
Description: Age
Count Code Label
----- ---- -----
0 -9 Not Asked
0 -8 Skipped
==
========
Name: block20_gender
Description: Gender
Count Code Label
----- ---- -----
485 1 Femme
515 2 Homme
0 8 Skipped
0 9 Not Asked

Description: Ingroup bias and outgroup evaluation - block16_9
Count Code Label ‘Chrétiens’
----- ---- -----
20 1 Très défavorable
44 2 Défavorable
397 3 Neutre
366 4 Favorable
173 5 Très favorable
0 8 Skipped
0 9 Not Asked
==
========
Name: block16_10
Description: Ingroup bias and outgroup evaluation - block16_10
Count Code Label ‘Musulmans’
----- ---- -----
195 1 Très défavorable
198 2 Défavorable
413 3 Neutre

150 4 Favorable
44 5 Très favorable
0 8 Skipped
0 9 Not Asked
==
========
Name: block16_11
Description: Ingroup bias and outgroup evaluation - block16_11
Count Code Label ‘Athées’
----- ---- -----
18 1 Très défavorable
31 2 Défavorable
447 3 Neutre
340 4 Favorable
164 5 Très favorable
0 8 Skipped
0 9 Not Asked

Name: block21_religion
Description: Religion
Count Code Label
----- ---- -----
553 1 Catholique
24 2 Protestante
35 3 Musulmane
10 4 Juive
3 5 Hindouiste, Bouddhiste
42 6 Agnostique
279 7 Athée
54 8 Autre.
0 98 Skipped
0 99 Not Asked
==
========
